# Association of pandemic precautions and *Staphylococcus aureus* in the NICU

**DOI:** 10.1017/ice.2025.10319

**Published:** 2025-12

**Authors:** Nora Elhaissouni, Abigail Arthur, Erica C. Prochaska, Elizabeth Colantuoni, B. Mark Landrum, Julia Johnson, Eili Klein, Aaron Milstone

**Affiliations:** 1 Division of Pediatric Infectious Diseases, Department of Pediatrics, Johns Hopkins University School of Medicine, Baltimore, MD, USA; 2 Department of Biostatistics, Johns Hopkins Bloomberg School of Public Health, Baltimore, MD, USA; 3 Department of Hospital Epidemiology and Infection Control, Johns Hopkins Hospital, Baltimore, MD, USA; 4 Division of Neonatology, Department of Pediatrics, Johns Hopkins University School of Medicine, Baltimore, MD, USA; 5 Department of International Health, Johns Hopkins Bloomberg School of Public Health, Baltimore, MD, USA; 6 Department of Emergency Medicine, Johns Hopkins University School of Medicine, Baltimore, MD, USA; 7 Division of Infectious Disease, Department of Medicine, Johns Hopkins University School of Medicine, Baltimore, MD, USA; 8 Johns Hopkins Howard County Medical Center, Columbia, MD, USA

## Abstract

In a retrospective cohort of 6363 neonates admitted to three NICUs, there was no reduction in *Staphylococcus aureus* acquisition when comparing pre- and post-pandemic incidence rates. While additional infection prevention practices introduced during the pandemic helped prevent SARS-CoV-2 transmission, these practices may not have reduced *S. aureus* transmission to infants.

## Introduction

Neonatal intensive care units (NICUs) serve vulnerable patient populations who are at increased risk of healthcare-associated infections (HAI). In this environment, *Staphylococcus aureus* (*S. aureus*) is a leading cause of HAIs, which increases morbidity and mortality.^1,2^ *S. aureus* transmission and outbreaks are common in the NICU despite rigorous infection prevention measures. Controlling *S. aureus* is challenging in the NICU because family, visitors, and health-care workers are often asymptomatically colonized and are known reservoirs for transmission.

Prior studies have shown that enhanced infection controls precautions, such as gowns and gloves, can reduce the transmission of bacteria in ICUs.^3^ During the pandemic, hospitals implemented enhanced infection control practices, including universal masking and limited visitation to prevent the spread of SARS-CoV-2. The pandemic provided an opportunity to assess whether masking and visitation restrictions may have decreased *S. aureus* transmission to infants in the NICU. Our objective was to explore the impact of pandemic infection control practices on *S. aureus* acquisition rates during compared to before the pandemic.

## Methods

We performed a retrospective cohort study including neonates admitted to three Johns Hopkins (JH) Health System NICUs (the JH Children’s Center, JH Bayview Medical Center and JH Howard County Medical Center) between July 2017 and December 2022. These NICUs had various room configurations (eg private, open bay, shared) yet consistent staffing ratios and hand hygiene adherence during the study period. We included all neonates admitted to the NICU for more than two calendar days, regardless of transfer status. All NICUs have a *S. aureus* control program of weekly nasal surveillance for all patients, admission nasal surveillance for outborn neonates, and decolonization of infants with *S. aureus*.^4^ All NICUs suspended 1) visitors exposed to or positive for SARS-CoV-2 throughout the duration of the pandemic and 2) sibling visitation. JHHS implemented mandatory universal staff and visitor masking in April 2020 through April 2023. This study was approved by the Johns Hopkins IRB with a waiver of informed consent.

The primary outcome was NICU-acquired *S. aureus* acquisition defined as having a nasal surveillance culture or a culture collected during clinical care (eg respiratory or blood culture) that grew *S. aureus* more than two days after NICU admission. We excluded neonates who had a positive culture within two days of admission. For infants who had positive surveillance and clinical cultures, we defined the outcome based on the timing of the first positive *S. aureus* culture. At-risk time was measured in patient days.

Descriptive statistics compared the characteristics of neonates admitted before and during pandemic periods. Outcomes were measured as monthly incidence rates (number of neonates per 1000 patient-days). Using Poisson regression models, we compared average monthly rate of NICU-acquired *S. aureus* acquisition before and during pandemic periods and fit an interrupted time series model (ITS) allowing for the immediate impact of the pandemic and potential changes in the trend of the monthly incidence. Statistical analyses were conducted using R Statistical Software v4.2.3.

## Results

During the study period, there were 6363 eligible infants after excluding 133 neonates who had a positive culture within two days of admission. Neonates before and during the pandemic period had similar proportions of neonates with birthweights <1500grams (33.3% and 35.6%, respectively), median length of NICU stay (12 and 11 days) and mortality rates (2.6% and 2.1%) (Table 1).

**Table 1.** Demographic and clinical characteristics of neonates in three NICUs between 2017 and 2022

	Pre-COVID^1^ (*N* = 3326)	Post-COVID (*N* = 3037)
Birthweight < 1500 g	1106 (33.3%)	1080 (35.6%)
Gestational age		
< 28 weeks	187 (5.6%)	148 (4.9%)
28–31 weeks	352 (10.6%)	243 (8.0%)
32–36 weeks	1120 (33.7%)	1018 (33.5%)
≥ 37 weeks	1667 (50.1%)	1628 (53.6%)
Mortality	88 (2.6%)	63 (2.1%)
Inborn	2872 (86.3%)	2593 (85.4%)
Median length of stay in days (IQR)	12 (6, 26)	11 (5, 24)
Neonates with positive *S. aureus* surveillance culture	492 (14.8%)	460 (15.1%)

^1^Neonates are classified in the pre- or post-pandemic period based on the date of their first nasal surveillance or clinical culture for those tested or admission date for those not tested.

Abbreviations: grams (g), interquartile range (IQR).

**Figure 1.**
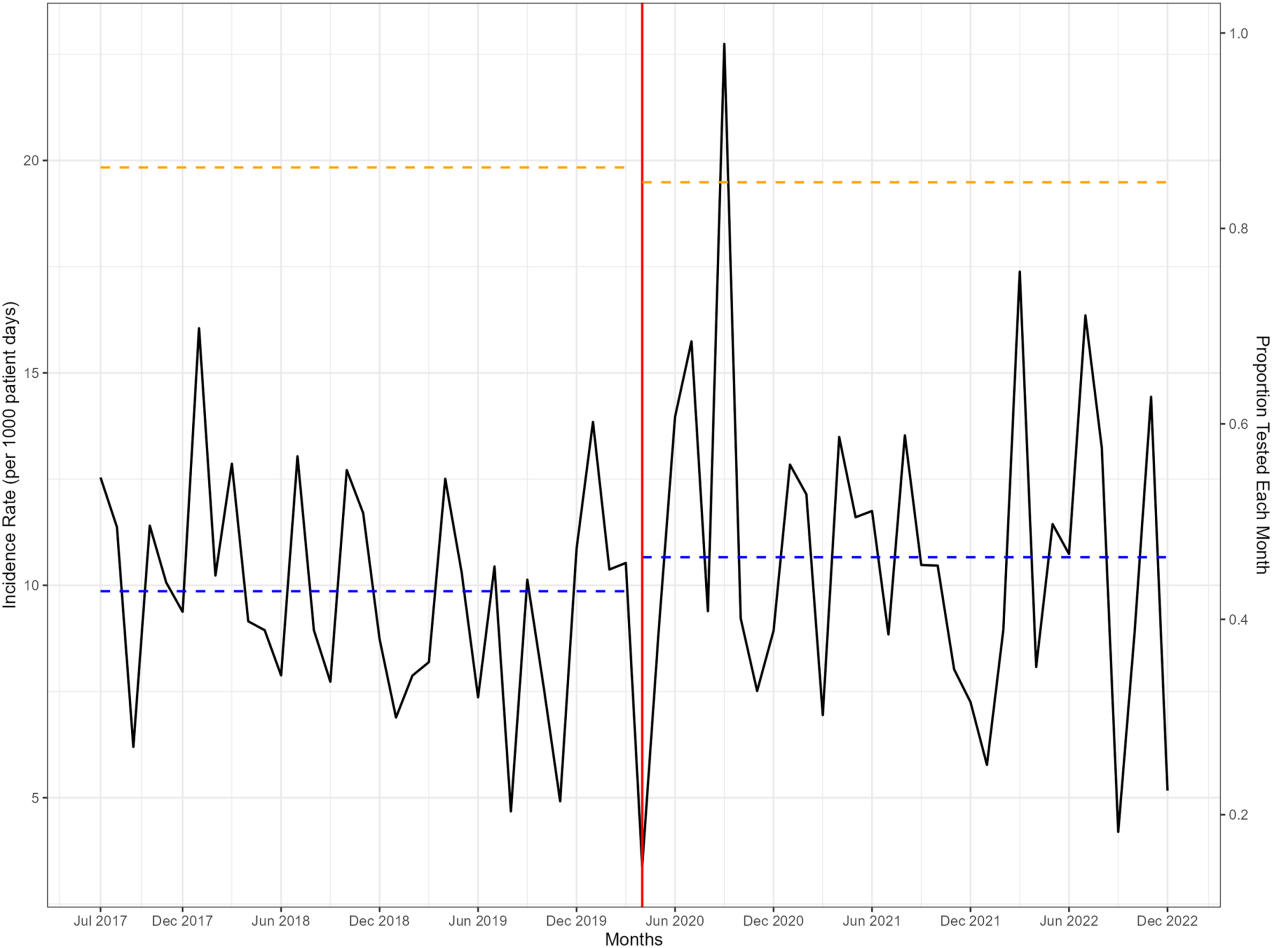
Monthly trend of unit-acquired *S. aureus* colonization, including both surveillance and clinical cultures, among infants in 3 NICUS before and after introduction of enhanced pandemic infection control measures from 2017–2022. Unit-acquired *S. aureus* acquisitions were defined as having either a first clinical or nasal surveillance culture that grew *S. aureus* more than two days after NICU admission. Incidence rates are *S. aureus* acquisitions per 1000 patient days. The dotted blue line represents the monthly average rate before the pandemic (9.86) and during the pandemic (10.66). The orange dotted line represents the monthly average proportion of infants who had a nasal surveillance culture collected before the pandemic (0.86) and during the pandemic (0.85) as a measure of adherence to surveillance testing.

During 93,456 at-risk days, 952 infants acquired *S. aureus* during their NICU admission through a positive nasal surveillance or clinical culture. Of these infants, 19 had a clinical culture that grew *S. aureus* prior to a positive nasal surveillance culture. Similar proportions of infants acquired *S. aureus* before and during pandemic periods (14.80% and 15.10%, respectively). The monthly incidence ranged from 3.38 to 22.74 colonizations per 1000 patient days (Figure 1). The monthly average proportion of infants who had a nasal surveillance culture collected before the pandemic (0.86) and during the pandemic (0.85) were similar. Across the entire study duration, the monthly proportion of infants who had a surveillance culture collected ranged from 0.74 to 0.93. The average monthly incidence of NICU-acquired *S. aureus* colonization was similar before and during the pandemic period (9.86 and 10.66 colonizations per 1000 patient days; incidence rate ratio [IRR] 1.08, 95% CI: 0.92–1.26). In the ITS model, there was a non-significant 23.1% (95% CI: 0.73–2.07) immediate increase in the colonization rate at the start of the pandemic (eTable 1).

## Discussion

*S. aureus* remains the most common cause of HAIs in infants in the NICU.^1^ Colonization is a well-known predisposing risk factor to infection.^5^ Current strategies to prevent *S. aureus* transmission and infections in NICUs include hand hygiene, environmental cleaning, and in some settings screening and decolonization.^6^ Enhanced infection prevention practices, such as universal masking and visitor restrictions, were introduced during the pandemic to prevent the spread of SARS-CoV-2.^7^ Our findings from three NICUs that maintained pre-pandemic prevention measures suggest that there was not a decrease in *S*. *aureus* acquisition following introduction of additional pandemic prevention measures. Our data are consistent with a previous study that reported higher NICU MRSA rates when instituting universal masking.^8^ Additionally, the lack of influence of changes in visitor polices on *S. aureus* incidence in the NICU was similarly consistent with another single-center study.^9^ Overall, these studies suggest that the increase in infection prevention strategies targeted to reduce the spread of SARS-CoV-2, did not reduce the spread of S *aureus* in the NICU.

Some important considerations may help explain why enhanced infection prevention practices may not reduce *S*. *aureus* transmission in the NICU. A recent study suggested that prolonged masking can change the nasal microbiota and increase *S. aureus* burden compared to mask-free periods.^10^ This higher colonization density may increase the risk for hand contamination when people touch their face and nose during times of prolonged masking.^10^ Regarding lack of impact of visitation strategies, parent visitation was not restricted in NICUs during the pandemic in the same fashion as in other hospital units. Visiting parents were required to wear masks, but if masking can increase *S. aureus* colonization burden,^10^ and parents are a known *S. aureus* reservoir,^4^ then pandemic precautions may have had an unintended consequence of temporarily increasing parent-to-child postnatal *S. aureus* transmission. Together, these findings suggest that although NICU policies should consider universal masking or visitor restrictions to prevent respiratory virus outbreaks, these strategies do not offer benefit to the control of *S. aureus* transmission in the NICU.

There were limitations to this analysis. Our study examined three NICUs in Maryland that consistently recommended *S. aureus* screening and decolonization, limiting the generalizability of the findings. Although there were additional infection prevention interventions used during the pandemic, implementation of and adherence to infection prevention interventions (eg masking) varied over time. While supply shortages may have reduced surveillance testing during the pandemic, the proportion of neonates screened remained consistent before and during pandemic periods. Further research is needed to identify other strategies to prevent *S. aureus* transmission in the NICU.

## Supporting information

Elhaissouni et al. supplementary material Elhaissouni et al. supplementary material

## Supplementary material

To view supplementary material for this article, please visit https://doi.org/10.1017/ice.2025.10319.
