# Supplementary material for: Association of pandemic precautions and Staphylococcus aureus in the NICU
Source: Infect Control Hosp Epidemiol. 2025 Oct 13;46(12):1262–4. doi: 10.1017/ice.2025.10319 (PMC12614451; doi:10.1017/ice.2025.10319)
Supplement: Elhaissouni et al. supplementary material [file S0899823X2510319Xsup001.docx]

**SUPPLEMENTAL ONLINE CONTENT**

| eTable 1 | **Results of interrupted time series analysis** |
| --- | --- |

This supplemental material has been provided by the authors to give readers additional information about their work.

**eTable 1: Results from the ITS model that assumes a Poisson distribution that controls for time trends and adjusts for patient days aimed at estimating the immediate pandemic impact and changes in the incidence rate over time in both the pre and post pandemic periods.**

| **Coefficient Type** | **Exponentiated Coefficient**  **(95% CI)** | **P Value** |
| --- | --- | --- |
| Relative change per month, pre-covid | 0.99 (0.98, 1.00) | 0.168 |
| Relative change at the start of the pandemic | 1.23 (0.73, 2.07) | 0.434 |
| Relative change per month, post-implementation | 1.0 (0.99, 1.00) | 0.33 |
| Relative Change per month post- vs pre- implementation | 1.002 (0.99, 1.02) | 0.795 |
